# Guideline-Adherent Hypertension in Children and Adolescents: A Multi-Institutional Database Analysis from Taiwan

**DOI:** 10.3390/jcm12134367

**Published:** 2023-06-28

**Authors:** Shao-Ju Chien, Lung-Chih Li, Hsiao-Ching Kuo, You-Lin Tain, Chien-Ning Hsu

**Affiliations:** 1 Division of Pediatric Cardiology, Kaohsiung Chang Gung Memorial Hospital and Chang Gung University College of Medicine, Kaohsiung 83301, Taiwan; csjdc@cgmh.org.tw; 2 Division of Nephrology, Department of Internal Medicine, Kaohsiung Chang Gung Memorial Hospital and Chang Gung University College of Medicine, Kaohsiung 83301, Taiwan; longee01@cgmh.org.tw; 3 Institute for Translational Research in Biomedicine, College of Medicine, Kaohsiung Chang Gung Memorial Hospital and Chang Gung University, Kaohsiung 83301, Taiwan; 4 Department of Pharmacy, Kaohsiung Chang Gung Memorial Hospital, Kaohsiung 83301, Taiwan; 5 Division of Pediatric Nephrology, Department of Pediatrics, Kaohsiung Chang Gung Memorial Hospital, Kaohsiung 83301, Taiwan; 6 College of Medicine, Chang Gung University, Taoyuan 33302, Taiwan; 7 School of Pharmacy, Kaohsiung Medical University, Kaohsiung 80708, Taiwan

**Keywords:** hypertension, blood pressure, children, adolescents, electronic health records, guidelines, outpatient, agreement

## Abstract

Background/Aims: Childhood-onset hypertension is associated with cardiovascular morbidity and adult mortality. This study aimed to assess guideline-adherent hypertension among Taiwanese youth and the agreement on hypertension between the 2017 American Academy of Pediatrics guidelines and the 2004 Fourth Report. Methods: In this cross-sectional study, we collected outpatient blood pressure (OBP) measurements obtained during routine care visits from a large healthcare delivery system between 2009 and 2018 to evaluate the rate of guideline-adherent hypertension and assess patient-related factors of pediatric hypertension. Results: In total, 12,469 children and adolescents who underwent three separate ≥3 OBP measurements over 33,369 person-years with a total of 95,608 BP measurements in an outpatient setting were analyzed. According to the 2017 American Academy of Pediatrics (AAP) guidelines, the rate of pediatric hypertension in the study setting, which included participants aged 1 to 17 years, ranged from 0.78 to 5.95 per 1000 persons. Although there was perfect agreement between the thresholds of the two guidelines for defining hypertension in the age groups of 1–7, 8–12, and 13–17 years (all κ statistic ≥ 0.85), the use of the AAP threshold classified more adolescents as having hypertension. Children and adolescents with hypertension often had complex chronic diseases and required substantial healthcare services in outpatient, emergency, and inpatient settings. Conclusions: The present study provides evidence of guideline-adherent pediatric hypertension and highlights the importance of regularly monitoring blood pressure to identify and manage hypertension in children and adolescents. Further research is required to determine the impact of new thresholds on the detection of target organ damage at a pediatric age.

## 1. Introduction

Hypertension contributes extensively to cardiovascular disease (CVD) mortality and disease burden globally [1]. Epidemiological and experimental evidence suggests that hypertension can develop in childhood or be determined perinatally [2,3]. Childhood hypertension progresses into adulthood and is closely associated with premature CVD and kidney disease [4]. Accordingly, the early detection and management of pediatric hypertension should be prioritized to reduce the global burden of hypertension and related diseases. When compared to adults, measuring hypertension in children is complicated and challenging [5]. A meta-analysis revealed that the global pooled pediatric hypertension occurrence was 4.0%, which steadily increased from 1.3% (1990–1999) to 6.0% (2010–2014) [6]. According to various studies, the reported incidence of hypertension in youth varies widely, ranging from 0.1% to 26.5% [6,7,8]. This large disparity may be attributed to the definition used, sample size, ethnicity, and differences between clinical and school-based settings [5].

For approximately two decades, the definition and treatment of hypertension in children were according to the blood pressure (BP) reference tables, which are based on sex, age, and height percentiles from the 2004 Fourth Report from the National Institute of Health’s National Heart, Lung, and Blood Institute [9]. In 2017, the American Academy of Pediatrics (AAP) published new BP guidelines for children. In comparison with the Fourth Report [9], it had simpler definitions of hypertension and static BP thresholds for adolescents aged ≥13 years matched with the new adult guidelines [10]. BP and vital signs are routinely measured among children and adolescents during care visits in the outpatient setting. However, elevated BP or hypertension may be inadequately diagnosed with specific disease codes by primary care providers [5,11]. This warrants the need to examine the rate of guideline-adherent pediatric hypertension in clinical practice settings.

The Chang Gung Research Database (CGRD) is a multi-institutional electronic health records (EHR) database derived from the network of Chang Gung Memorial Hospitals (CGMHs) in Taiwan. The CGMHs provided approximately 11% of health services reimbursed by Taiwan’s National Health Insurance in 2020. It includes over 8.7 million outpatient and emergency department visits and 274,500 hospital admissions [12]. Several studies have assessed pediatric hypertension using the BP data recorded in the United States EHR systems [11,13], yet the utility of outpatient BP (OBP) measurements and the diagnostic BP threshold for pediatric hypertension in Taiwan remain unknown. To fill this knowledge gap, this novel study used OBP data derived from the CGRD to assess the guideline-defined hypertension and patient-related factors of pediatric hypertension. Additionally, we examined the agreement on hypertension between the 2017 AAP guidelines and the 2004 Fourth Report to improve the recognition of hypertension among Taiwanese youth.

## 2. Materials and Methods

### 2.1. Study Design and Data Source

This was a retrospective cross-sectional study in which the participants underwent multiple OBP measurements from 2009 to 2018. The participants included children and adolescents who received outpatient care from pediatric specialists at the CGMHs. Available patient-level EHR data were retrieved from the CGRD, which covered medical encounters, diagnosis, prescription and dispensing, procedures, and laboratory results [14,15]. Among the 7,900,047 patient visits from 1 January 2009 to 31 December 2018, we identified 105,900 participants aged 1–20 years who had at least one measurement of both systolic BP (SBP) and diastolic BP (DBP) at the same outpatient visit.

### 2.2. Study Cohort

**Figure 1:**
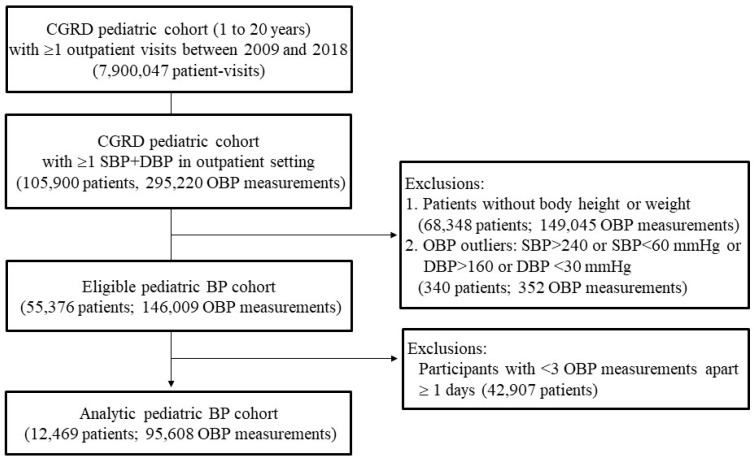
Study population. CGRD, Chang Gung Research Database; SBP, systolic blood pressure; DBP, diastolic blood pressure; OBP, outpatient blood pressure.

Body weights, heights, and OBP were ascertained from the file “Blood pressures in the outpatients” in the CGRD. To understand age-specific hypertension, the participants whose heights were not measured during the OBP monitoring visits were excluded [16]. The quality of the weight- and height-for-age data in the 1-year age interval was examined to exclude incorrect or outlier growth data in the EHR system using an algorithm derived from the 2007 WHO reference table (<−6 standard deviation [SD] and >+6 SD of Z-scores for weight and height) [17]. After excluding missing data (*n* = 68,348) and invalid measures (<0.1% of all observations), 55, 376 participants were included in the eligible study cohort. Second, the distributions of the SBP- and DBP-for-age data in the 1-year interval were examined, and the BP data outliers were excluded (SBP > 240, SBP < 60, DBP > 160, or DBP < 30 mmHg) (*n* = 340) [18]. Lastly, to ensure the validity of hypertension, the participants with less than 3 separate OBP measurements at least 1 day apart over the study period were excluded (*n* = 42,907). Further, 12,469 children and adolescents with ≥ three separate visits [16], each with OBP and height measurements, were included for BP classification (Figure 1).

### 2.3. Blood Pressure Measurement and Classification

OBP was measured in seated children and adolescents who had been resting for at least 3–5 min by registered nurses during the outpatient visits. The registered nurses who work in the pediatric outpatient setting have advanced knowledge and skills in the general care of children and adolescents. An oscillometric sphygmomanometer was used, as it is the preferred BP measurement device for the CGMH network. The cuff width was a minimum of 40% of the arm circumference measured at the midpoint between the shoulder and elbow and 80–100% of the length [19]. The BP monitors are annually checked by the Department of Medical Equipment in the CGMH network by following a standard procedure to ensure proper functioning.

Age–sex–height SBP and DBP percentiles were calculated for the participants aged between 1 and 17 years based on the reference tables of the 2004 Fourth Report and the 2017 AAP guidelines, respectively [9,10]. For the participants aged ≥18 years, hypertension was defined similarly to that of adults with an SBP ≥ 140 and/or DBP ≥ 90 mmHg [20]. The same SBP and DBP values were assigned to one of the three BP categories, hypertension, prehypertension/elevated BP, and normal BP, according to the 2004 Fourth Report and the 2017 AAP guidelines, respectively (Table 1). Using three separate occasions within 1 year of age, hypertension was defined as ≥three SBP and/or ≥three DBP measurements meeting the BP thresholds, similar to the definition of prehypertension/elevated BP in both guidelines [9,10].

### 2.4. Covariates

Along with the measured height and weight, the body mass index (BMI) was calculated as body weight (kg) divided by the square of height (m). The BMI percentile for specific age- and sex-value was determined based on the Taiwan reference table [21], which adopted the definitions from the growth charts for children and adolescents by the Centers for Disease Control and Prevention [22]. The participant was defined as overweight if their BMI was between the 85th and 95th percentile specific to sex and age, obesity was described as BMI ≥ 95th percentile, and underweight was <5th percentile [22].

Medical history was assessed based on the data from one year before the OBP measured date using the Pediatric Medical Complexity Algorithm (PMCA) Version 2.0, which employs conservative criteria [23]. Disease conditions were identified based on at least two International Classification of Diseases 9th/10th Revision (ICD-9/10) diagnosis codes for the disease in outpatient/emergency department settings or at least one code for the disease at hospital discharge [23]. The PMCA was developed by the Seattle Children’s Hospital Center of Excellence on Quality of Care Measures for Children with Complex Needs, USA. The algorithms classify children aged 0 to 18 years into three levels of medical complexity based on ICD codes for health conditions in both claims and hospital discharge data: complex chronic disease (CD), non-complex CD, and without CD [23]. It also included 18 body systems and a progressive condition (Supplementary Table S1). The conditions of progressive disease are associated with deteriorating health and increased mortality risk. In addition, the number of outpatient visits, emergency department visits, and hospitalizations within one year before the OBP measured was assessed to investigate their association with childhood-onset hypertension.

### 2.5. Statistical Analyses

Guideline-based hypertension and elevated or normal BP were identified among children and adolescents who had repeated OBP measurements within 1 year of age. The rate of hypertension in the group of 1–7, 8–12, 13–17, and 18–20 years was calculated as the number of hypertension cases divided by the total number of individual participants at risk for the disease in the age period. The four age groups were not mutually exclusive. To explore the trend of pediatric hypertension in the EHR system between 2009 and 2018, the annual rate of hypertension was calculated as the number of hypertension cases divided by the total number of individual participants in outpatient visits in a given calendar year. The rates of pediatric hypertension are presented as per 1000 persons.

Agreement on hypertension between the two guidelines was assessed separately in groups: 1–7, 8–12, and 13–17 years. Cohen’s kappa (κ) estimate with a 95% confidence interval (CI) was calculated for the reliability between the two versions of the guidelines for pediatric hypertension, and κ ≤ 0.2 was considered as no agreement, 0.21–0.40 as fair, 0.41– 0.60 as moderate, 0.61–0.80 as substantial, and 0.81–1.00 as almost perfect agreement [24]. The adjusted odds ratio with a 95% confidence interval (aOR [95% CI]) was estimated using a conditional logistic regression model to assess the association between patient factors and hypertension (vs. elevated and normal BP) in each age group. The statistical analyses were performed using SAS software (version 9.4; Cary, NC, USA). A probability value of *p* < 0.05 was considered significant between comparison groups.

### 2.6. Ethics

The study was approved by the Institutional Review Board of Chang Gung Medical Foundation in Taipei, Taiwan (IRB No: 202100993B0). Patient informed consent was waived owing to the retrospective nature of the study, and all procedures were performed according to the guidelines of the Declaration of Helsinki.

## 3. Results

### 3.1. Pediatric Hypertension

Over the 10-year study period, 12,469 youths who underwent ≥1 OBP and height measurements within 1 year of age over 33,369 person-years with a total of 95,608 OBP measurements were analyzed. Adolescents aged 13 years and older accounted for 74% of the total cumulative person-years followed by 8–12 years (16.76%) and 1–7 years (8.62%).

According to the 2017 AAP guidelines (Table 2), the rate of pediatric hypertension was 5.95 per 1000 persons among the older adolescents aged 13–17 years (*n* = 218,368), 2.24 per 1000 persons among the younger adolescents aged 8–12 years (*n* = 255,615), and 0.78 per 1000 persons among the younger children aged 1–7 years (*n* = 495,962). The 2017 AAP guidelines defined hypertension at a higher rate in the older children and adolescent groups than in the younger children group.

Table 2 shows a perfect agreement (κ > 0.8) between the thresholds of the 2004 Fourth Report and 2017 AAP guidelines for defining hypertension in all age groups. However, the agreement was slightly lower among the older adolescents aged 13–17 years based on Cohen’s kappa estimate (κ = 0.85; 95% CI, 0.84–0.87). This is because the adolescents aged ≥13 years had a threshold value of hypertension of ≥130 and/or ≥80 mmHg in the 2017 AAP version instead of the percentile (≥95th +5 mmHg) reference table of the 2004 Fourth Report.

**Figure 2:**
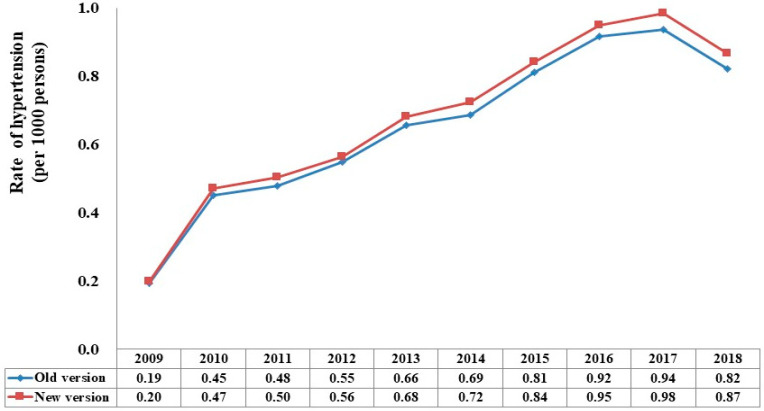
Annual rate of pediatric hypertension among children and adolescents in the CGRD from 2009 to 2018. CGRD, Chang Gung Research Database; Old version: 2004 Fourth Report; New version: 2017 AAP guidelines.

Figure 2 depicts an increasing trend of the annual rate of pediatric hypertension between the 2004 Fourth Report and the 2017 AAP guidelines over the study period. Although the rate of hypertension was slightly higher in the 2017 AAP guidelines than in the 2004 Fourth Report, the difference in the annual rates was considered small (absolute difference: 0.01/1000 persons in 2009 and 0.04/1000 persons in 2017).

### 3.2. Patient Factors Associated with Childhood-Onset Hypertension

Of the 12,469 children and adolescents, 50.06% were boys (*n* = 6272). The percentage distribution based on age was 42% at 13–17 years (*n* = 5288), 28% at 18–20 years (*n* = 3491), 18% at 8–12 years (*n* = 2262), and 11% at 1–7 years (*n* = 1428) at their first visit with OBP measurement in the entire study period (Table S1). The overall rate of insulin-dependent diabetes mellitus (IDDM) was higher than non–insulin-dependent diabetes mellitus (NIDDM) (4.33% vs. 2.58%, respectively) in this study cohort, and IDDM was more prevalent (11.76%) in younger children (<13 years) than NIDDM (2.48%) (Table S1).

Using the 2017 AAP guidelines, Table 3 presents the distribution of characteristics of the study cohort according to BP classification during the follow-up period. The mean BMI was higher in the participants with hypertension (24.51 ± 6.37) than in the participants with elevated BP (22.75 ± 4.87) and normal BP (20.92 ± 4.88). For NIDDM, the rate increased in the children with elevated BP and hypertension in comparison with the children with normal BP, which was similar with the rate of obesity (Table 3). Within a one-year baseline period, the participants with hypertension utilized more health services through outpatient visits, emergency departments, and hospitalizations than those with elevated and normal BP. For instance, the children and adolescents with hypertension had double the number of outpatient visits (11.58 ± 12.45) than those with normal BP (6.55 ± 6.47).

We used the PMCA to demonstrate the status of comorbidity in the different BP groups and discovered that 53.69% of person-years with hypertension had complex chronic disease, and 34.59% had a progressive disease condition (Table 3). Table 4 demonstrates that obesity and more healthcare services in outpatients and hospitalizations were significantly associated with hypertension in the participants aged ≥8 years (all *p* < 0.05). The odds of having a cardiac disease associated with hypertension were higher in the older ages of 18–20 years (aOR, 5.58 [1.75–17.74]) followed by 8–12 years (aOR, 4.33 [1.11–16.89]) and 13–17 years (aOR, 3.03 [1.47–6.26]). Among the participants aged 13–17 years, pulmonary/respiratory (aOR, 2.62 [1.09–6.26]) and progressive status (aOR, 2.88 [1.23–6.73]) were associated with hypertension. These results indicate that baseline health status and comorbidities carry different risks of hypertension in different age groups.

## 4. Discussion

This study presents the rates of childhood-onset hypertension in a large sample of a pediatric outpatient setting based on approximately 0.8 million children and adolescent visits in Taiwan. Several important findings were summarized in the present study. First, the rate of pediatric hypertension ranged from 0.78 to 5.95 per 1000 persons between 1 and 17 years of age in the pediatric cohort in Taiwan. Second, the annual rate of pediatric hypertension gradually increased from 2009 to 2018. Third, the agreement was high (κ ≥ 0.85) on the threshold values between the 2004 Fourth Report and 2017 AAP guidelines for defining pediatric hypertension across all pediatric age groups. Finally, in comparison with children and adolescents with normal BP, children and adolescents with hypertension suffered from complex chronic diseases and required extensive healthcare services in the outpatient, emergency department, and inpatient settings.

The estimate of hypertension using OBP readings in the present study was lower than the estimates from national survey studies [25,26]. Unlike population surveys using single-time BP measurements (single or average multiple measurements on one occasion) [25,26], the BP readings in our study were independently obtained from each occasion of BP measurement during the multiple outpatient visits. Hypertension may be underestimated in EHR systems when BP is not monitored or when patients are not followed up in the given EHR system. However, our study results suggested that the burden of pediatric hypertension was approximately two-fold higher than that in a population-based study using ICD-9 codes that define hypertension between 2000 and 2013 in Taiwanese youths [27]. These results suggest that pediatric hypertension is underdiagnosed and underestimated in Taiwan.

Consistent with the evidence derived from a systematic review of childhood hypertension at the global level [6], we found that the occurrence of hypertension was high during puberty and peaked at the end of puberty. Pediatric hypertension rapidly increased from 2009 to 2018. These findings are consistent with the trends among children and adolescents in the United States, which increased from 2011–2014 to 2015–2018 [28].

The 2017 AAP guidelines agreed with the 2004 Fourth Report, which defined hypertension and detected more adolescents with hypertension among Taiwanese youth. These findings are supported by those in different pediatric populations [29,30,31]. However, whether the 2017 AAP guidelines are more sensitive and decisive for the detection of target organ damage in Taiwanese youth warrants further clarification. Clinicians should be aware that recalculating percentiles using the 2017 AAP guidelines and higher BP percentiles yield more cases of hypertension in the pediatric population. The differences in the BP classifications presented in the present study should be carefully considered as clinicians track and understand the need for longitudinal BP control in patients.

It is worth noting that the 2016 European Society of Hypertension (ESH) guideline for pediatric hypertension adopted the 2004 Fourth Report BP reference tables but recommended the use of adult BP cut-points for adolescents at least 16 years of age [32]. According to a systematic review and meta-analysis, the AAP guidelines identified more cases of pediatric hypertension than the ESH and Fourth Report guidelines [31]. Moreover, the meta-analyses based on three observational studies revealed that the thresholds of these guidelines (AAP or Fourth Report/ESH guidelines) showed similar performance in the detection of childhood onset left ventricular hypertrophy [31].

All three current guidelines recommend repeated BP measurements in outpatient settings to confirm the clinical observation of the first event with at least three separate visits [9,10,32]. However, the protocol for the diagnosis of pediatric hypertension may result in the dropout of cases and is challenging to apply in practice [33,34]. Once hypertension is detected (patients with a single high BP measurement obtained in a practice setting), the guidelines recommend a second visit to confirm hypertension [10,35]. Home BP measurements using an automated device or ambulatory BP monitoring are advised to be used as a diagnostic tool for hypertension in children and adolescents [10,32].

We evaluated the complexity of chronic comorbidities and organ system disorders and their association with childhood-onset hypertension. Our study results support the idea that children with complex chronic diseases have a greater need for healthcare services, such as inpatient care, than those without [35]. Secondary causes of hypertension are common in the pediatric population. Children identified with hypertension using PMCA taxonomy were more likely to have a comorbid condition of cardiac, endocrinological, metabolic, and kidney diseases in the age groups of 8–12 and 13–17 years. Consistent with previous literature, the rate of IDDM was relatively higher than NIDDM in the entire study cohort, and it was more prevalent in younger children (<13 years of age) [36]. Because NIDDM has shown associations with overweight/obesity in some pediatric populations [32], further research is required to specify whether diabetes mellitus was a comorbidity among hypertensive patients aged 8–20 years or the cause of hypertension. These types of chronic conditions were consistent with the guidelines recommended for the management of secondary hypertension in patients of pediatric age [33]. In addition, our results highlight other chronic diseases, such as neurological conditions, which were also prevalent among children aged 8–12 years as well as mental health conditions among groups of 8–12 and 18–20 years. They also imply that the OBP measurements were taken by pediatric specialists during outpatient visits for children with specific disease conditions.

Pediatric guidelines recommend routine BP measurement in children from 3 years of age; in children with low birth weight, premature birth, and congenital defects of the kidney, heart and other organs, they should begin assessment earlier than 3 years of age [10,32]. Although the proportion of children aged 1–7 years was lower than that of older children (>8 years of age) in this study, there was no difference in the number of children identified with hypertension between the two guidelines. Younger children had a greater rate of complex chronic disease as compared to the other age groups, suggesting that secondary hypertension should be addressed in future studies.

These study findings of guideline-adherent hypertension during childhood and adolescence support the need for interventional studies for the development of age-specific hypertension screening and diagnosis strategies. Identifying youth with an accelerated increase in hypertension from age 8 to 17 years will allow health professionals to initiate preventive interventions early in life, since improvement in hypertension treatment and lifestyle factors (including activity and dietary modifications) have been shown to reduce the rate of cardiovascular disease and lead to better hypertension control [37,38].

This study has several strengths compared with prior pediatric hypertension studies, including the large number of OBP readings obtained from routine pediatric care settings, not limited to referral or a hypertension clinic. Our study assessed multiple OBP values with at least three separate visits, which was more accurate than a one-time measurement in a practice setting. In addition, this is one of the few studies evaluating childhood-onset hypertension across different children and adolescent age groups.

This study has some limitations. First, due to the retrospective observational nature of the evidence, the rate of pediatric hypertension in children and adolescents who received repeated follow-up OBP measurements in the study setting could be biased toward secondary hypertension. The exclusions of fewer OBP measurements and missing height data increased the validity of guideline-defined hypertension in the pediatric age; however, it may weaken the representativeness of the study cohort. Furthermore, we cannot rule out potential biases due to the wider adoption of BP assessment guidelines in practice, which results in the identification of more hypertension cases in recent years. Second, BP readings were obtained using a manual oscillometric sphygmomanometer. Although this method is widely used in our practice, BP measurements taken by health professionals can vary between office visits as recognized by the guidelines of the American Heart Association and American College of Cardiology for adults [39]. Third, we relied on OBP readings recorded in the structural data format of the CGRD to identify abnormal BP. The OBP measurements in the text notations of the EHR were not available for research, and their influence on our current findings is uncertain. These limitations may misclassify participants into various BP categories, though the overall rate of pediatric hypertension and patient factors in this study were consistent with those of other studies using an EHR [40]. Additionally, our results from a large pediatric cohort across a wide age range may not apply to specific populations and different healthcare systems.

## 5. Conclusions

Measuring the burden of hypertension in the pediatric population is challenging. We used EHR data from CGMHs to assess pediatric hypertension and the characteristics of children and adolescents with hypertension, elevated BP, or normative BP. Although the agreement metrics between the 2017 AAP and 2004 Fourth Report guidelines were high, further elucidation is required on whether the new thresholds for hypertension can advance the identification of target organ damage in children and adolescents. Despite the advances made in the field of pediatric hypertension, our results highlight the importance of early detection and management of hypertension by measuring BP regularly in outpatient workflows for all children and adolescents to prevent hypertensive target organ injury.

## Figures and Tables

**Table 1:** Classification of office blood pressure measurements.

2004 Fourth Report *		2017 AAP for Children 1–13 y *		2017 AAP for Children ≥13 to <18 y	
Classification	SBP/DBP percentile	Classification	SBP/DBP percentile	Classification	SBP/DBP
Normal BP	<90th	Normal BP	<90th	Normal BP	<120/80 mmHg
Prehypertension	90th to < 95th or (≥120 and/or ≥80 mmHg, even if <90th, up to <95th)	Elevated BP	≥90th to <95th or (≥120 and/or ≥80 to <130 and/or <80 mmHg)	Elevated BP	≥120 and/or ≥80 to< 130 and/or <80 mmHg
Hypertension	≥95th + 5 mmHg	Hypertension	≥95th to <95th +12 mmHg or (≥130/80 mmHg)	Hypertension	≥130 and/or ≥80 mmHg

* SBP/DBP percentile was determined based on the reference tables of the guidelines; In participants aged ≥18 years, hypertension was defined as ≥140/89 mmHg; American Academy of Pediatrics (AAP); systolic blood pressure (SBP); diastolic blood pressure (DBP).

**Table 2:** Comparison of pediatric hypertension defined by 2004 and 2017 versions of the guidelines in three age groups.

Age Group, Year	Number of Participants *	2004 Fourth Report		2017 AAP Guidelines		Differences		
		Number of Cases	Event Rate/ 1000 Persons	Number of Cases	Event Rate/ 1000 Persons	Kappa (95% CI) **		Agreement (%) ***
1–7	495,962	375	0.76	387	0.78	0.98	(0.97–0.99)	99.58
8–12	255,615	523	2.05	573	2.24	0.94	(0.94–0.96)	99.11
13–17	218,368	1185	5.43	1300	5.95	0.85	(0.84–0.87)	97.44

American Academy of Pediatrics (AAP). * Number of participants was the number of individual participants who had ≥1 outpatient blood pressure measurements within 1 year of age in the range of age group. ** Cohen’s Kappa estimate (κ) > 0.8 indicates perfect agreement. *** Agreement (%) between the two guidelines was calculated as the following: number of cases + number of non-cases agreed in both guidelines/total participants.

**Table 3:** Characteristics of the study cohort by blood pressure classification using the 2017 version of the guidelines.

	Overall	Hypertension		Elevated BP		Normal BP		*p*-Value
Person-years *	33,369	3963		2145		27,261		
Age group, year, n (%)								<0.0001
1–7	2875	387	(9.77)	111	(5.17)	2377	(8.72)	
8–12	5594	573	(14.46)	219	(10.21)	4802	(17.61)	
13–17	13,010	1300	(32.80)	816	(38.04)	10,894	(39.96)	
18–20	11,890	1703	(42.97)	999	(46.57)	9188	(33.70)	
Person-years *	31,357	3836		2060		25,461		
BMI, mean (±SD)		24.51	(±6.37)	22.75	(±4.87)	20.92	(±4.88)	
Underweight	4128	232	(6.05)	168	(8.16)	3728	(14.64)	<0.0001
Normal	16,471	1391	(36.26)	1018	(49.42)	14,062	(55.23)	
Overweight	4359	615	(16.03)	364	(17.67)	3380	(13.28)	
Obesity	6399	1598	(41.66)	510	(24.76)	4291	(16.85)	
Person-years *	33,351	3958		2144		27,249		
Outpatient visit, mean (±SD) **		11.58	(±12.45)	9.12	(±7.28)	6.55	(±6.47)	<0.0001
1–3	11,197	478	(12.08)	378	(17.63)	10,341	(37.95)	
4–7	11,041	1297	(32.77)	781	(36.43)	8963	(32.89)	
8–12	6197	944	(23.85)	520	(24.25)	4733	(17.37)	
>12	4916	1239	(31.30)	465	(21.69)	3212	(11.79)	
Emergency department, mean (±SD) **		0.70	(±1.79)	0.53	(±1.34)	0.39	(±1.04)	<0.0001
None	24,177	2409	(60.86)	1376	(64.18)	20,392	(74.84)	
1	6612	1095	(27.67)	578	(26.96)	4939	(18.13)	
≥2	2562	454	(11.47)	190	(8.86)	1918	(7.04)	
Hospitalization, mean (±SD) **		0.66	(±1.36)	0.49	(±1.23)	0.26	(±0.88)	<0.0001
None	26,047	2391	(60.41)	1463	(68.24)	22,193	(81.45)	
1	5671	1153	(29.13)	525	(24.49)	3993	(14.65)	
≥2	1633	414	(10.46)	156	(7.28)	1063	(3.90)	
Person-years *	33,351	3958		2144		27,249		
PMCA, n (%) **								<0.0001
Without chronic disease	12,032	660	(16.68)	578	(26.96)	10,794	(39.61)	
Non-complex Chronic disease	10,644	1173	(29.64)	759	(35.40)	8712	(31.97)	
Complex Chronic disease	10,675	2125	(53.69)	807	(37.64)	7743	(28.42)	
Cardiac	5011	920	(23.24)	250	(11.66)	3841	(14.10)	<0.0001
Craniofacial	184	26	(0.66)	11	(0.51)	147	(0.54)	0.6278
Dermatological	41	6	(0.15)	6	(0.28)	29	(0.11)	0.0754
Endocrinological	4679	831	(21.00)	421	(19.64)	3427	(12.58)	<0.0001
IDDM	2509	296	(7.47)	228	(10.63)	1985	(7.28)	<0.0001
NIDDM	1072	350	(8.83)	119	(5.55)	603	(2.21)	<0.0001
Gastrointestinal	2274	406	(10.26)	196	(9.14)	1672	(6.14)	<0.0001
Genetic	468	88	(2.22)	39	(1.82)	341	(1.25)	<0.0001
Genitourinary	657	118	(2.98)	38	(1.77)	501	(1.84)	<0.0001
Hematological	526	129	(3.26)	48	(2.24)	349	(1.28)	<0.0001
Immunological	1195	299	(7.55)	94	(4.38)	802	(2.94)	<0.0001
Malignancy	1469	250	(6.32)	142	(6.62)	1077	(3.95)	<0.0001
Mental health	3124	522	(13.19)	236	(11.01)	2366	(8.68)	<0.0001
Metabolic	1416	426	(10.76)	112	(5.22)	878	(3.22)	<0.0001
Musculoskeletal	606	126	(3.18)	51	(2.38)	429	(1.57)	<0.0001
Neurological	4996	829	(20.94)	390	(18.19)	3777	(13.86)	<0.0001
Ophthalmological	852	184	(4.65)	80	(3.73)	588	(2.16)	<0.0001
Otologic	556	86	(2.17)	53	(2.47)	417	(1.53)	0.0001
Pulmonary/Respiratory	1658	247	(6.24)	128	(5.97)	1283	(4.71)	<0.0001
Renal	1676	537	(13.57)	118	(5.50)	1021	(3.75)	<0.0001
Progressive	6294	1369	(34.59)	460	(21.46)	4465	(16.39)	<0.0001

BP, blood pressure; (N)IDDM, (Non-) Insulin-dependent diabetes mellitus; * Person-years were the sum of each participant who had ≥1 outpatient blood pressure measurements within 1 year of age multiplied by the follow-up years. ** Healthcare service utilization and Pediatric Medical Complexity Algorithm (PMCA) were identified within 1 year prior to the first blood pressure measured of the age at outpatient visit.

**Table 4:** Patient factors associated with pediatric hypertension.

		1–7 Y			8–12 Y				13–17 Y		18–20 Y		
		aOR	(95% CI)	*p*-Value	aOR	(95% CI)	*p*-Value	aOR	(95% CI)	*p*-Value	aOR	(95% CI)	*p*-Value
**BMI (vs. normal)**													
	Underweight	1.19	(0.47−3.04)	0.7175	0.81	(0.25−2.65)	0.7330	0.76	(0.32−1.78)	0.5228	0.54	(0.17−1.68)	0.2877
	Overweight	1.43	(0.66−3.06)	0.3633	1.85	(0.94−3.62)	0.0735	1.32	(0.80−2.19)	0.2834	1.23	(0.74−2.06)	0.4322
	Obesity	1.69	(0.78−3.65)	0.1853	2.38	(1.04−5.44)	0.0407	2.72	(1.36−5.45)	0.0049	2.07	(0.97−4.40)	0.0603
**Outpatient visit (vs. 1–3)**													
	4–7	1.70	(0.73−3.95)	0.2166	1.86	(0.96−3.61)	0.0679	2.08	(1.33−3.25)	0.0013	3.00	(1.88−4.81)	<.0001
	8–12	2.19	(0.88−5.44)	0.0911	2.57	(1.24−5.32)	0.0114	2.40	(1.44−4.00)	0.0008	2.92	(1.70−5.03)	0.0001
	>12	3.72	(1.33−10.40)	0.0122	3.07	(1.35−6.97)	0.0074	2.94	(1.61−5.35)	0.0004	2.87	(1.57−5.25)	0.0006
**Emergency department**		1.40	(0.77−2.52)	0.2689	1.27	(0.75−2.15)	0.3807	0.99	(0.67−1.48)	0.9772	1.46	(0.96−2.23)	0.0769
**Hospitalization**		2.62	(1.43−4.82)	0.0019	1.80	(1.08−3.01)	0.0247	1.42	(0.98−2.05)	0.0675	2.29	(1.42−3.69)	0.0007
PMCA *													
	Cardiac	3.17	(0.82−12.29)	0.0950	4.33	(1.11−16.89)	0.0348	3.03	(1.47−6.26)	0.0027	5.58	(1.75−17.74)	0.0036
	Endocrinological	3.84	(0.16−92.87)	0.4078	2.73	(0.47−15.95)	0.2643	1.93	(0.95−3.93)	0.0710	1.94	(0.84−4.50)	0.1236
	Genitourinary	0.17	(0.02−1.74)	0.1358	0.49	(0.07−3.36)	0.4713	2.21	(0.32−15.29)	0.4228	0.23	(0.02−2.98)	0.2596
	Hematological	1.77	(0.28−11.30)	0.5448	1.43	(0.03−61.06)	0.8533	2.36	(0.42−13.28)	0.3287	0.47	(0.10−2.19)	0.3380
	Mental health	0.87	(0.42−1.78)	0.6958	1.70	(0.67−4.28)	0.2646	1.19	(0.48−2.97)	0.7141	1.71	(0.66−4.45)	0.2692
	Metabolic	6.33	(0.55−72.60)	0.1381	1.26	(0.19−8.63)	0.8124	1.18	(0.48−2.91)	0.7223	0.83	(0.34−2.04)	0.6842
	Musculoskeletal	8.57	(0.60−122.82)	0.1137	0.30	(0.04−2.28)	0.2449	0.28	(0.04−2.19)	0.2230	2.31	(0.57−9.36)	0.2430
	Neurological	0.84	(0.21−3.43)	0.8094	3.26	(0.63−16.97)	0.1602	1.52	(0.57−4.07)	0.4061	1.92	(0.72−5.12)	0.1913
	Ophthalmological	1.48	(0.37−5.92)	0.5762	7.03	(0.78−63.74)	0.0828	2.57	(1.02−6.48)	0.0460	1.97	(0.70−5.57)	0.2008
	Otologic	0.69	(0.10−4.62)	0.7054	1.27	(0.18−8.88)	0.8103	0.72	(0.15−3.57)	0.6872	1.47	(0.39−5.48)	0.5680
	Pulmonary/Respiratory	0.56	(0.14−2.20)	0.4067	1.03	(0.41−2.55)	0.9581	2.62	(1.09−6.26)	0.0306	0.70	(0.18−2.71)	0.6009
	Renal	0.83	(0.13−5.46)	0.8475	1.11	(0.25−4.84)	0.8916	1.64	(0.63−4.29)	0.3145	0.82	(0.17−4.06)	0.8059
	Progressive	1.48	(0.55−3.96)	0.4355	1.41	(0.15−12.86)	0.7630	2.88	(1.23−6.73)	0.0149	1.97	(0.88−4.44)	0.1017

aOR: adjusted odds ratio; CI: confident interval; * The number of patients with a craniofacial, dermatological, gastrointestinal, genetic, immunological, malignancy in the PMCA (Pediatric Medical Complexity Algorithm) was too small to show significance in some age groups and was not shown in the table.

## Data Availability

The data that support the findings of this study are available from the corresponding author upon reasonable request.

## Supplementary Materials

The following supporting information can be downloaded at: https://www.mdpi.com/article/10.3390/jcm12134367/s1, Table S1: Characteristics of children and adolescents at first time of blood pressures measured in the entire study period.
