# Supplementary material for: Guideline-Adherent Hypertension in Children and Adolescents: A Multi-Institutional Database Analysis from Taiwan
Source: J Clin Med. 2023 Jun 28;12(13):4367. doi: 10.3390/jcm12134367 (PMC10342897; doi:10.3390/jcm12134367)
Supplement: Supplementary file 1 [file jcm-12-04367-s001.zip › jcm-2435157-supplementary.pdf]

Table S1 Characteristics of children and adolescents at the first blood pressure measurement in the entire study period

	Overall	1-7 y	8-12 y	13-17 y	18-20 y
Number of patient evaluated, n	12469	1428	2262	5288	3491
**Hypertension (2017 AAP guideline), n (%)**					
Hypertension	1498 (12.01)	222 (15.55)	210 (9.28)	488 (9.23)	578 (16.56)
Elevated BP	832 (6.67)	52 (3.64)	78 (3.45)	326 (6.16)	376 (10.77)
Normal BP	10139 (81.31)	1154 (80.81)	1974 (87.27)	4474 (84.61)	2537 (72.67)
**Sex ,n (%)**					
Boys	6242 (50.06)	821 (57.49)	1251 (55.31)	2609 (49.34)	1561 (44.71)
Girls	6227 (49.94)	607 (42.51)	1011 (44.69	2679 (50.66)	1930 (55.29)
Number of patients evaluated, n	12368	1426	2262	5199	3481
**BMI, Mean (±SD)**	21.11 (±5.09)	16.35 (±2.65)	19.60 (±4.64)	22.01 (±4.73)	22.71 (±5.22)
Underweight, n (%)	1657 (13.40)	102 (7.15)	213 (9.42)	697 (13.41)	645 (18.53)
Normal, n (%)	6675 (53.97)	935 (65.57)	1274 (56.32)	2737 (52.64)	1729 (49.67)
Overweight, n (%)	1640 (13.26)	192 (13.46)	314 (13.88)	684 (13.16)	450 (12.93)
Obesity, n (%)	2396 (19.22)	197 (13.81)	461 (20.38)	1081 (20.79)	657 (18.87)
Number of patient evaluated, n	12458				
**PMCA**, n (%)					
Without chronic disease	5592 (44.89)	393 (27.54)	809 (35.78)	2659 (50.3)	1731 (49.68)
Non-complex chronic disease	3661 (29.39)	556 (38.96)	813 (35.96)	1447 (27.37)	845 (24.25)
Complex chronic disease	3205 (25.73)	478 (33.5)	639 (28.26)	1180 (22.32)	908 (26.06)
Cardiac	1417 (11.37)	268 (18.78)	278 (12.3)	552 (10.44)	319 (9.16)
Craniofacial	57 (0.46)	18 (1.26)	9 (0.4)	18 (0.34)	12 (0.34)
Dermatological	10 (0.08)	1 (0.07)	4 (0.18)	3 (0.06)	2 (0.06)
Endocrinological	1326 (10.64)	89 (6.24)	264 (11.68)	599 (11.33)	374 (10.73)
IDDM	539 (4.33)	60 (4.20)	171 (7.56)	236 (4.46)	72 (2.06)
NIDDM	321 (2.58)	12 (0.84)	37 (1.64)	168 (3.18)	104 (2.98)
Gastrointestinal	683 (5.48)	37 (2.59)	112 (4.95)	254 (4.81)	280 (8.04)
Genetic	121 (0.97)	27 (1.89)	31 (1.37)	38 (0.72)	25 (0.72)
Genitourinary	199 (1.60)	39 (2.73)	55 (2.43)	64 (1.21)	41 (1.18)
Hematological	193 (1.55)	22 (1.54)	27 (1.19)	79 (1.49)	65 (1.87)
Immunological	370 (2.97)	55 (3.85)	51 (2.26)	141 (2.67)	123 (3.53)
Malignancy	494 (3.97)	36 (2.52)	72 (3.18)	194 (3.67)	192 (5.51)
Mental health	1100 (8.83)	227 (15.91)	342 (15.13)	333 (6.3)	198 (5.68)
Metabolic	379 (3.04)	18 (1.26)	56 (2.48)	189 (3.58)	116 (3.33)
Musculoskeletal	190 (1.53)	18 (1.26)	22 (0.97)	66 (1.25)	84 (2.41)
Neurological	1621 (13.01)	468 (32.8)	346 (15.3)	481 (9.1)	326 (9.36)
Ophthalmological	242 (1.94)	53 (3.71)	38 (1.68)	85 (1.61)	66 (1.89)

Otologic	216 (1.73)	27 (1.89)	33 (1.46)	96 (1.82)	60 (1.72)
Pulmonary/Respiratory	623 (5.00)	131 (9.18)	157 (6.94)	191 (3.61)	144 (4.13)
Renal	445 (3.57)	56 (3.92)	108 (4.78)	173 (3.27)	108 (3.1)
Progressive	1825 (14.65)	232 (16.26)	341 (15.08)	733 (13.87)	519 (14.9)

AAP, American Academy of Pediatrics; BP, blood pressure; BMI, body mass index; SD, standard deviation; (N)IDDM, (Non-) Insulin-dependent diabetes mellitus; PMCA, Pediatric Medical Complexity Algorithm
